# Comparison of bulk milk antibody and youngstock serology screens for determining herd status for Bovine Viral Diarrhoea Virus

**DOI:** 10.1186/s12917-016-0797-2

**Published:** 2016-08-26

**Authors:** Richard E. Booth, Joe Brownlie

**Affiliations:** Royal Veterinary College, Hawkshead Lane, North Mymms, Hertfordshire, AL9 7TA UK

**Keywords:** Bovine Viral Diarrhoea Virus, Herd screen, Herd status, Bulk milk antibody, Youngstock check test

## Abstract

**Background:**

This paper examines the use of Bulk Milk antibody (BM Ab), Youngstock (YS) serology (Check Tests) and Bulk Milk PCR (BM PCR) for determining the presence or absence of animals persistently infected (PI) with Bovine Viral Diarrhoea Virus (BVDV) within a herd. Data is presented from 26 herds where average herd sizes were 343 and 98 animals for dairy and beef units respectively. Seventeen herds had sufficient data to analyse using Receiver Operating Characteristic (ROC) and probability curves enabling calculation of the sensitivity and specificity of BM Ab and YS Check tests for determining the presence of PI animals within herds in this dataset.

**Results:**

Using BM Ab to screen a herd for the presence of PI animals, achieved a herd level sensitivity and specificity of 80.00 % (44.39–97.48 %) and 85.71 % (42.13–99.64 %) respectively (95 % confidence intervals quoted). Sensitivity and specificity of YS Check Tests at a cut off of 3/10 Ab positive YS were 81.82 % (48.22–97.72 %) and 66.67 % (22.28–95.67 %) respectively (95 % confidence interval). These results were achieved by comparing the screening tests to whole herd PI searches that took place 1–19 months after the initial screen with a mean interval of 8 months. Removal of this delay by taking BM samples on the day of a whole herd test and simulating a YS Check Test from the herd test data produced improvements in the reliability of the Check Tests. BM Ab sensitivity and specificity remained unchanged. However, the Check Test sensitivity and specificity improved to 90.9 % (58.72–99.77 %) and 100 % (54.07–100 %) respectively (95 % confidence interval) at a cut of off 2.5/10 Ab positive animals. Our limited BM PCR results identified 5/23 dairy farms with a positive BM PCR result; two contained milking PIs, two had non-milking PIs and another had no PIs identified.

**Conclusions:**

Delaying a PI search following an initial herd screen decreased the diagnostic accuracy and relevance of our results. With careful interpretation, longitudinal surveillance using a combination of the techniques discussed can successfully determine farm status and therefore allow changes in BVDV status to be detected early, thus enabling prompt action in the event of a BVDV incursion.

## Background

Bovine Viral Diarrhoea Virus (BVDV) is an economically important pestivirus recognised for causing infertility, immunosuppression and, as a consequence, high levels of secondary disease in cattle herds worldwide [1–7]. The losses associated with BVDV infection are well documented and the disease is commonly quoted to cost the UK farming industry £40 million per year primarily as a consequence of sub-optimal fertility and immunosuppression [8]. At the individual animal level, the most recent published estimates of losses due to BVDV infection are €32 and €63 per cow per year in beef and dairy systems respectively within the Irish cattle sector [9]. Similar figures of £37 per cow per year exist for beef suckler herds in the UK [10], but less detail is reported at the individual cow level within the UK dairy sector. Significantly, a number of European countries have recognised the losses caused by BVDV and undertaken national eradication; whilst Norway, Sweden, Finland and Denmark have eradicated it [11, 12], other countries e.g. Austria, Switzerland, Germany, Belgium, Ireland and Scotland are in the process of eradication [11, 13–15]. The Scottish and Irish programmes include control measures with regulations to prevent the sale of persistently infected (PI) carrier animals [13, 14]. This will impact on further eradication efforts throughout England and Wales since clearly it would be beneficial for these programmes to be compatible with those underway in Scotland and Ireland in order to facilitate trade.

The persistently infected (PI) animal is infected as a foetus in the first trimester of pregnancy and born immunotolerant to the infecting strain of BVDV; thereafter becoming a lifelong shedder of the virus [16, 17] excreting large quantities of BVDV in all body secretions [18, 19]. Control of PI animals is critical to the successful control of BVDV transmission both within and between herds. Lindberg et al. 1999 [20] drew conclusions from epidemiological studies of BVDV and stated that “in practice, a herd is not infected until one or more PIs have been established” and, in addition to this, “BVDV cannot persist in a herd where contacts between PI animals and susceptible animals in early pregnancy do not occur”. This highlights the pivotal role of PI animals in the epidemiology of BVDV and the need to both identify and cull them as part of any successful BVDV eradication programme. The more rapid the culling of PIs from infected herds, the greater the health and productivity advantage.

Two approaches to BVDV eradication have been utilised by those European countries that have programmes in place. In some, often where seroprevalence was deemed to be high, the decision was made at the outset to test directly for PI animals at the national level without establishing status at the herd level first i.e. Switzerland and Ireland [14, 21]. In both programmes, specialised ear tags were used to collect ear notch tissue samples for testing for BVDV antigen or RNA. Within the Swiss programme, the aim was to test the whole cattle population [21] whereas, in Ireland it has been compulsory to test newborn animals as part of the official tagging process from January 1^st^ 2013 onwards [14]. In contrast, the Scandinavian and Scottish programmes have elected firstly to establish individual herd status and then proceed with eradication in infected herds [13, 20]. At the outset of these eradication programmes, effective herd level screening was essential in order to distinguish between BVDV-infected (contains PI animals) and BVDV-free herds (no PI animals present). This allows resources to be focussed on either identification and culling of PI animals or on surveillance and protection. It is always essential that effective biosecurity is implemented to prevent the re-introduction of infection [20, 22, 23]. Depending upon local circumstances and, in particular cattle density, enhanced biosecurity may be combined with vaccination to protect against herd re-infection [24]; this is especially important where biosecurity does not meet the stringent standards outlined in the Cattle Health Certification Standards (CHeCS) technical document [25].

Within England and Wales, many farmers will not know their current BVDV status and it is hoped that this manuscript will provide practitioners with further advice on the use of appropriate herd level diagnostics to determine whether PI animals are likely to be present. Current recommendations for UK herd BVDV accreditation are documented in the CHeCS technical document [25]. Two options are permissible for herd BVD accreditation. The first involves screening successive calf crops for BVDV antigen and accreditation is achieved following negative test results over a 2 year period. The second method involves antibody (Ab) screening of bulk milk samples and youngstock (YS) cohorts (commonly referred to as ‘Check Tests’ or ‘Spot Tests’) in order to accurately assess herd BVDV status. BM Ab has played a major role in the control of cattle diseases since the mid-1980s [26] and was widely used within the Scandinavian BVDV control programmes to establish dairy herd status. The CHeCS technical document recommends the use of quarterly BM Ab screening tests at the point of establishing and monitoring herd status although it recognises a positive result may occur in a herd that has been historically infected; at this point the screening of first lactation animals may provide a more up to date status of the dairy herd [25]. The recommendations within CHeCS for YS screening in the UK are largely based on the work conducted in Denmark and the USA by Houe 1992, Houe 1994 and Houe et al. 1995 [27–29]. The first study determined that herds ranging in size from 96 to 324 animals (with a mean of 135) could be grouped into BVDV infected and BVDV-free herds by testing only three or five YS 6–18 months of age [27]. In a further study, forty-two herds were investigated ranging in size from 40 to 157 animals and in a detailed survey of seven of these herds, low levels of YS seroprevalence were found to correlate with the absence of PI animals (0–1/10 YS Ab positive at the spot test) and high levels of YS seroprevalence correlated with the presence of PIs (8–10/10 YS Ab positive at the spot test) [28]. An additional test available for milk samples is bulk milk PCR (BM PCR) which enables the screening of bulk tank milk for the presence of PI animals. This allows the user to establish the status of the milking animals that contribute to the sample, but not necessarily the entire herd. Initially BM PCR was reported to be sensitive enough to detect one PI animal in 160 milking animals [30], but more recently this has increased to an upper limit of 300 recommended by many diagnostic laboratories [31] with further laboratories reporting a higher diagnostic sensitivity allowing this upper limit to increase further to 1000 animals (Karen Bond, National Milk Laboratories, Personal Communication).

This paper examines and discusses the use and practical implications of BM Ab, YS Check Tests and BM PCR for determining the presence or absence of PI animals using data collected from 26 working UK farms involved in a pilot BVDV eradication programme [32] where average herd sizes were 343 (interquartile range: 224 to 431) and 98 (interquartile range: 58 to 121) animals for the dairy and beef units respectively.

## Methods

Farm recruitment began in April 2006 and was largely complete by April 2007. The farms involved and further details of their recruitment are described by Booth & Brownlie 2012 [32]. Farm ID numbers used throughout this manuscript are consistent with those used by Booth & Brownlie 2012 [32] and Booth et al. 2013 [33] enabling cross referencing between publications. All samples were collected by veterinary surgeons as part of the routine infectious disease surveillance on the farms involved and the herd owners gave signed consent for the data collected to be reported anonymously.

Upon recruitment to the pilot BVDV eradication programme, each farm was screened to determine herd status (BVDV-infected/BVDV-free) using a combination of BM Ab, BM PCR and YS Check Tests. Throughout this study, Check Tests consisted of 10 animals of approximately 9 months of age (range 6–18 months, with a preference of 9–12 months) from each separate management group on the farm. If fewer than ten animals were available in the age range required for the Check Test then all available animals were sampled. All samples were submitted to and tested by the Animal Health and Plant Agency (AHPA) via AHPA Starcross. BM samples were submitted in universal containers with Bronopol preservative (AHPA, Weybridge) and blood samples were submitted in heparinised vacutainers. AHPA test codes for the BVDV BM Ab ELISA and PCR were TC0123 and TC0709 respectively. All BM Ab results were reported as Optical Density (OD) ratios and interpreted such that OD ratios <0.1 = negative, 0.1–0.35 = low positive, 0.35–0.7 = mid positive and >0.7 = high positive. BM PCR results were reported as either positive or negative. All blood samples tested for Ab (AHPA test code TC0390) were reported as OD ratios and interpreted such that <0.2 = negative and > 0.2 = positive. Further details of the laboratory tests used in this paper including test sensitivity, specificity and further details regarding cut off values have been described previously by Booth and Brownlie 2012 [32]. Since the data presented in this paper was collected the TC0390 and TC0123 ELISA tests have been superseded and it should be noted that the stated cut-off values are not applicable to the ELISA tests currently offered by the AHPA.

Following each initial screen, the results were assessed in order determine herd exposure and whether BVDV was likely to be active on the farm and thus whether PI animals were likely to be present. For the purposes of this project, active infection based upon the initial screens was defined cautiously as any herd with >1/10 Ab positive YS and/or a positive BM PCR. Where evidence of exposure to BVDV indicated that the infection was likely to be active, a whole herd test (WHT) was performed where the entire herd was blood sampled in order to identify any PI animals present. Due to the longevity of the immune response following field infection with BVDV [7, 34] high BM Ab levels alone were not deemed sufficient to advise WHTs in the absence of a positive BM PCR or Ab positive YS and farms with this combination of initial results were subjected to further regular surveillance (described below). In beef herds, initial herd screens consisted solely of Check Tests. The laboratory tests and testing regimes used for WHTs have been described previously by Booth and Brownlie 2012 [32]. If identified, PIs were either culled or retained on the farm of origin; this was the farmer’s choice. Where PI animals were identified, the recommendation was to cull them - sale of PI animals (unless direct to slaughter) was not permitted as a condition of membership of this study.

In herds where initial screening did not indicate an active BVDV infection, the farmer was given the choice of either blood sampling the whole herd to confirm the accuracy of the screen, or implementing regular herd surveillance. If a WHT was selected, once it was confirmed that there were no PI animals within the herd, the farm implemented regular herd surveillance from that point onwards. Within this study, regular herd surveillance consisted of BM Ab testing (at least quarterly), BM PCR testing (at least yearly) and Check Tests performed at least once a year. In herds undergoing regular surveillance, if active infection (as defined above) was suspected at any point, surveillance frequency was initially increased meaning that a further Check and BM Ab test was performed within 4–8 weeks of the preceding one. In the event that active infection was still suspected, a WHT was performed to search for PIs. Again, in beef herds, surveillance consisted solely of Check Tests.

All farms with data reported in this manuscript were monitored for at least 3 years (the majority joined the study in 2006/2007 and remained members until it ended in 2014), thus the data collected accurately reflects the number of PI animals present on each of the study farms over the period presented. On farms that did not undergo WHTs because ongoing herd surveillance indicated that there was no source of BVDV exposure, the assumption was made that no PI animals were present at any time.

For herds that underwent WHTs and had full BM Ab and Check Test results from their initial screen, Receiver Operating Characteristic (ROC) curves were generated in SPSS (SPSS Inc., Chicago) to compare the ability of BM Ab and Check Tests to determine the presence of PI animals in a herd. The optimal cut-off value, sensitivity and specificity of each test was then determined. Using logistic regression, predicted probability curves were produced in ‘R’ (The R Foundation for Statistical Computing, 2008) using “xyplot <lattice>” for BM Ab and YS Check Tests to illustrate how the probability of identifying a herd containing a PI(s) alters according to the results of the initial screens.

For some herds there were considerable delays between the initial herd screen and the WHT. This consisted of time needed to persuade the farmer to undertake a WHT and agreement on a suitable date (at times, harvesting or silaging meant a considerable delay was incurred). In order to investigate the effect of this delay on the accuracy and relevance of the initial herd screens, BM Ab and BM PCR results from the day of the WHT were collected. In order to simulate a YS Check Test as if performed on the day of the WHT, ten Ab results from YS aged as close to 9–12 months as possible were randomly selected from those sampled during the WHT. The YS chosen were selected using a random number generator in Microsoft Excel (2007) which was used to select ten animals from a list of those present on the day of the WHT arranged in age order starting with the animal closest to 9 months of age on the day of the WHT. Where insufficient animals were present within the 9–12 month range, the range was expanded to 7–13 months. The ROC and probability curve calculations described above were then repeated using data from the day of the WHT in effect removing any delay between conducting a herd screen and acting on the results.

## Results

Twenty-six farms (Table 1) from the original 41 study members described by Booth and Brownlie 2012 [32] had results for both an initial herd screen to determine BVDV status and either whole herd tests (WHT) to identify PI animals or sufficient surveillance (described above) to determine that PI animals were unlikely to be present during the study period. Individual details of the 26 farms analysed in this manuscript are shown in Table 1. The group consisted of three beef herds and 23 dairy herds. Average herd sizes were 343 (interquartile range: 224 to 431) and 98 (interquartile range: 58 to 121) animals for the dairy and beef units respectively. Of the farms involved, the majority, 20/26 (77 %), were vaccinating against BVDV using either Pregsure BVD (Pfizer Animal Health, UK) or Bovilis BVD (MSD Animal Health, Milton Keynes, Buckinghamshire) (Table 1). All vaccines were administered according to the datasheet recommendations from the manufacturer with the primary course completed at an appropriate time prior to first service; a minimum of 14 days and 4 weeks for Pregsure and Bovilis BVD respectively.

**Table 1:** Study Farm Details

Farm ID	Dairy/Beef	Vaccinating over study period?	Suspicion of active infection following initial screen?^^^	Was whole herd testing performed?	PIs present at WHT	Total number of PIs identified	Time between initial herd screen and WHT (Months)	Age range of random sample (Months)	Herd size at recruitment
1	Dairy	Y*	Y	Y	2	3	11	9–12	349
2	Dairy	N	Y	Y	0	12	7	9–12	542
3	Dairy	Y*	N	Y	0	0	10	9–12	450
5	Dairy	Y*	Y	Y	2	2	7	9–12	527
6	Beef	Y*	N	N	-	0	-	-	61
7	Dairy	Y*	Y	Y	0	0	9	9–12	381
8	Dairy	Y*	Y	Y	0	0	3	11.5–13	381
11	Dairy	Y*	N	N	-	0	-	-	217
13	Dairy	N	N	N	-	0	-	-	201
15	Dairy	Y*	N	Y	1	1	9	7.7–12	633
17	Beef	Y*	N	N	-	0	-	-	180
18	Dairy	Y*	Y	Y	4	6	9	10–12.6	307
19	Dairy	N	Y	Y	1	1	5	9–12	214
20	Dairy	Y*	Y	Y	3	3	4	9–12	309
21	Beef	N	N	N	-	0	-	-	54
24	Dairy	N	N	N	-	0	-	-	286
25	Dairy	Y*	N	Y	0	0	9	9.3–13.5	300
26	Dairy	Y*	Y	Y	2	3	5	8.5–12.3	170
27	Dairy	Y*	N	Y	1	1	11	9–12	192
28	Dairy	Y^+^	Y	Y	0	0	12	9–12	335
29	Dairy	Y^+^	Y	Y	0	0	12	8–11	412
30	Dairy	Y^+^	N	N	-	0	-	-	360
37	Dairy	Y^+^	Y	Y	3	5	2	10.1–12.3	402
38	Dairy	Y^+^	Y	Y	2	2	3	8.3–10.9	542
39	Dairy	N	N	Y	0	0	19	9–12	143
40	Dairy	Y*	Y	Y	5	9	1	9–12	230
				Mean			8		315
				Max			19		633
				Min			1		54

Details of the farms analysed in this manuscript: Farm type, vaccination status (* vaccinating using Pregsure BVD and ^+^ vaccinating using Bovilis BVD), Suspicion of active infection upon initial screening (^^^ according to the cautious initial interpretation), Total number of PIs identified during the study, Whether a whole herd test was performed, Total number of PIs identified at the WHT (where performed), The delay between initial screening and WHT if conducted, Age range of animals selected for the random screening at the WHT and herd size at recruitment. Farm numbers can be cross referenced with Booth and Brownlie 2012 [32] and Booth et al. 2013 [33]

With the exception of Farms 2, 5 and 15, all farms reported in this manuscript reared homebred youngstock on the farm where they were born.Farms 2, 5 and 15 removed animals at, or shortly after, weaning to separate units where they were reared to return to the home farm for first calving. Farms 5 and 15 only reared their own animals at these separate sites. Farm 2 reared bull calves and non-replacement heifers on the main farm unit as beef animals, however they also utilised a heifer rearer for their replacement dairy heifers. These animals were moved to the heifer rearer after weaning where they were kept with animals from another farm with no attempt to separate them. Once at service age, a bull was introduced and the heifers returned to the main farm to calve.

**Fig. 1:**
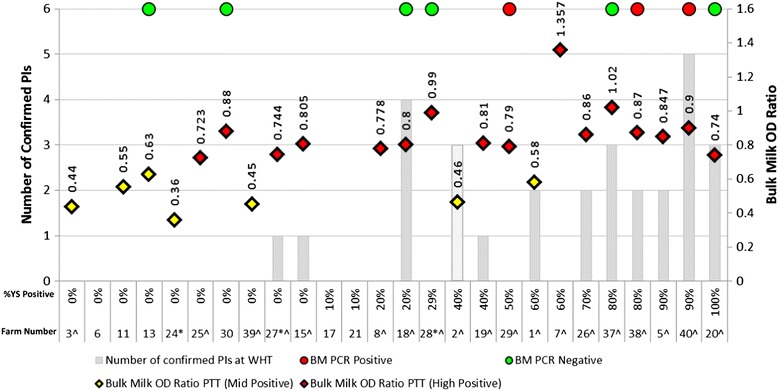
The results of the initial herd screens of the 26 study farms. The figure illustrates the results of the initial herd screens for the 26 farms presented in this manuscript. ^ indicates the 19 herds where WHTs were performed. The left y-axis displays the number of PIs identified at the WHT (where performed) depicted for each farm by the solid bars - the Farm 2 bar is hashed to represent in-utero PI animals. For the 7 farms that did not undertake WHTs because their regular surveillance did not indicate exposure, it was assumed that no PIs were present. The right y-axis displays the BM Ab OD ratio. Along the x -axis, each column represents an individual farm stating both the percentage of antibody positive youngstock (YS) at the Check Test (* all farms except Farms 24, 27 & 28 submitted ten animals who presented 9, 8 & 7 youngstock respectively) and the Farm number. (Graph Key attached to the bottom of Fig. 1)

The results of the initial herd screens for all 26 farms are presented in Fig. 1 alongside the number of PIs found in each herd at the WHT. The total number of PIs identified on each farm and the initial number found at the WHT are shown in Table 1. With the exception of Farm 2, in all herds where PIs were identified, at least one of those PIs was present from the outset of the study. For Farm 2, the initial herd screens identified 40 % seroconversion at the YS Check Test (conducted at the heifer rearing unit) and a mid-positive BM Ab titre. The seropositive youngstock initiated a WHT on this farm at both the main farm where the milking animals were kept and the heifer rearing unit. Permission was not given to test the animals at the heifer rearing unit that were mixed with those from Farm 2. No PIs were identified at the Farm 2 WHT in year 1, however, due to the seroconversion noted in the youngstock, newborn animals were tested as they were born onto the farm and the first PI was identified in year 2 of the study as a ‘trojan’ PI born to an non-PI heifer returning from a heifer rearing unit where mixing with animals from multiple sources had occurred [32]. Indeed the subsequent two PIs detected on Farm 2 were also born to non-PI heifers returning from the heifer rearer. For these reasons, within the ROC and probability curve analysis described below, Farm 2 was treated as positive when analysing Check Test results, but negative in the BM analyses. Following the birth of the third PI the adult herd had been infected long enough to produce a fourth PI from a 3^rd^ lactation animal. In total, twelve PIs were confirmed on this farm (with two antigen tests four weeks apart) with a further 9 that either died or were culled before they could be confirmed with a second antigen test [32].

Seven of the 26 study herds (the three beef units; Farms 6, 17 & 21 and four of the dairy units; Farms 11, 13, 24 &30) returned regular surveillance results which indicated that they were unlikely to have been exposed to a source of BVDV infection. These herds were excluded from the statistical analysis because they did not undertake WHTs. The remaining 19 herds undertook WHTs however two of these, Farms 27 and 28, were excluded from the statistical analysis since only 7 & 8 YS respectively were available in the appropriate age range for their initial screens. Therefore, of the 26 study herds, a total of 17 were suitable for ROC and probability curve analysis since they had both 10 YS and a BM Ab sampled at initial recruitment and also conducted a WHT. Furthermore, it is only these 17 farms that contribute to the sensitivity and specificity results stated below.

### Bulk milk antibody analysis

**Fig. 2:**
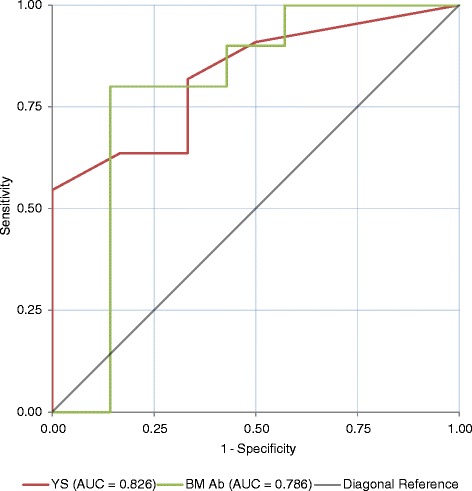
ROC curves demonstrating the performance of BM Ab and YS Check Tests as predictors of the presence of a PI animal(s) where delays occurred between the initial herd screen and the WHT. The ROC curves were generated from the seventeen dairy farms in Fig. 1 with full initial herd screens and WHT results. YS = Youngstock check testing and BM Ab = Bulk milk antibody testing. Farm 2 is considered negative for BM Ab analysis and positive for YS analysis. AUC = Area under curve. Coordinates of the curves are given in Table 2

**Fig. 3:**
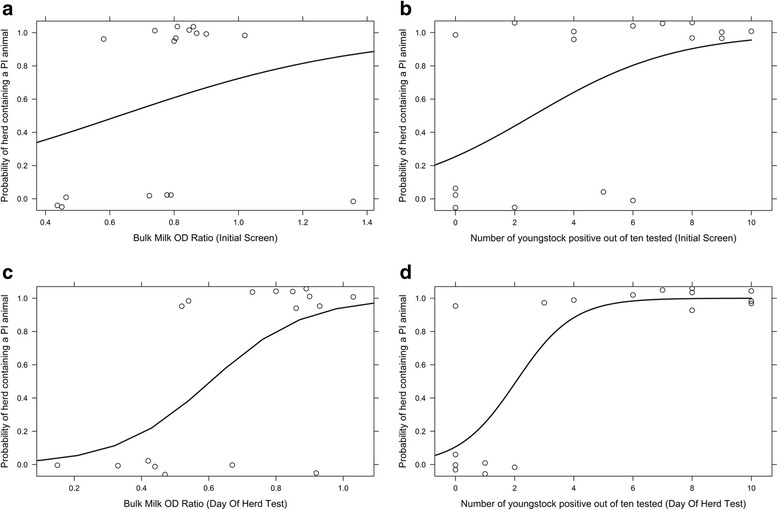
Predicted Probability Curves indicating the probability of a study herd containing a PI animal with the stated BM Ab and YS Check Test results. Data points demonstrating PI presence are ‘jittered’ around 0 or 1 on the y axis to show the number of farms that contribute to the curve at each point, **a** BM Ab OD Ratio and the probability of identifying a herd containing a PI animal with delays between performing the BM test and the WHT; **b** Number of YS positive out of the ten tested and the probability of identifying a herd containing a PI animal with delays between performing the Check Test and the WHT; **c** BM Ab OD Ratio and the probability of identifying a herd containing a PI animal with no delay between performing the BM test and the WHT; **d** Number of YS positive out of the ten tested and the probability of identifying a herd containing a PI animal with no delays between performing the Check Test and the WHT. Farm 2 is considered negative for BM Ab analysis and positive for YS Check Test analysis

BM Ab titres on the 23 dairy farms observed in Fig. 1 range from mid to high positive. Interestingly, high positive BM Ab results occur on six farms where no PIs were identified (Farms 7, 8, 25, 28, 29 & 30) and mid positive results occur on two farms where PIs were identified (Farms 1 and 2). On Farm 2 however, it should be noted that the dairy herd would not have had contact with PI animals by this point. Of the dairy farms observed in Fig. 1, none began the study with low positive or negative BM Ab titres, yet 11/23 (48 %) had no PI animals identified. The BM Ab ROC curve in Fig. 2 and its coordinates (Table 2) indicate that the optimal cut-off point for distinguishing whether or not a herd contained a PI animal using the BM Ab titre is achieved at an OD ratio of 0.7950 units with a sensitivity and specificity of 80.00 % (44.39–97.48 %) and 85.71 % (42.13–99.64 %) respectively (95 % confidence intervals quoted). Of the 17 farms analysed in Fig. 2, using a BM OD ratio cut off of 0.7950 there were two false negative farms (i.e. would be declared free of PIs when they were present) (Farms 1 & 20) and one false positive (i.e. would be thought to contain PI animals when there were none present) (Farm 7). For Farm 2, the BM Ab OD ratio of 0.46 units and interpretation that there were not PI animals present can be considered correct for the adult dairy herd given that it had not been exposed to PI animals at this point. Fig. 3a displays the predicted probability curve for BM Ab OD ratio calculated for the 17 farms that were used for the ROC curve analysis. The curve shows a gradual but limited increase in probability of detecting a PI as BM Ab OD ratio increases.

**Table 2:** Coordinates of the ROC curves in Fig. 2

Test result variables	Positive if greater than or equal to a or b^#^	Sensitivity	1 - Specificity
^a^Number of Youngstock antibody positive out of ten tested with delays between the YS screen and whole herd test	−1.00	1.000	1.000
	1.00	.909	.500
	3.00	.818	.333
	4.50	.636	.333
	5.50	.636	.167
	6.50	.545	0.000
	7.50	.455	0.000
	8.50	.273	0.000
	9.50	.091	0.000
	11.00	0.000	0.000
^b^Bulk milk antibody OD ratio with delays between the BM Ab screen and whole herd test	−.5630	1.000	1.000
	.4440	1.000	.857
	.4575	1.000	.714
	.5225	1.000	.571
	.6520	.900	.571
	.7315	.900	.429
	.7590	.800	.429
	.7840	.800	.286
	.7950	.800	.143
	.8025	.700	.143
	.8075	.600	.143
	.8285	.500	.143
	.8535	.400	.143
	.8650	.300	.143
	.8850	.200	.143
	.9600	.100	.143
	1.1885	0.000	.143
	2.3570	0.000	0.000

^#^ The smallest cutoff value is the minimum observed test value minus 1, and the largest cutoff value is the maximum observed test value plus 1. All the other cutoff values are the averages of two consecutive ordered observed test values

### Check test analysis

In Fig. 1, antibody positive YS identified at the initial Check Test are presented as a percentage of the total tested since Farms 24, 27 and 28 only presented 9, 8 and 7 YS respectively in the required age range. All other farms had ten YS available for the Check Test. When observing the number of antibody positive YS identified on initial herd screening in Fig. 1, the number of antibody positive YS increases with the likelihood of identifying a herd containing a PI animal. Using the Check Test results alone and the original cut-off (defined above) of >1/10 Ab positive animals signifying a BVDV infected farm results in two false negative farms (Farms 15 & 27) and four false positives (Farms 7, 8, 28 & 29). Analysing the Check Test results from the seventeen dairy herds that undertook WHTs, with Farm 2 included as infected since three heifers amongst the group sampled were carrying in-utero PIs at this point, the coordinates (Table 2) of the ROC curve in Fig. 2, indicate two potential cut off points. The first uses a cut off of 5.5/10 YS BVDV Ab positive (thus interpreting results with either ≤5 YS or ≥6 YS as herds that are negative and positive respectively) and returns a sensitivity and specificity of 63.64 % (30.79–89.07 %) and 83.33 % (35.88–99.58 %) respectively (95 % confidence intervals quoted). The second cut off is at 3/10 YS BVDV Ab positive and returns a sensitivity and specificity of 81.82 % (48.22–97.72 %) and 66.67 % (22.28–95.67 %) respectively (95 % confidence intervals quoted). The second cut off of 3/10 YS BVDV Ab positive may be preferred marginally due to the slightly higher sensitivity and thus smaller likelihood of mis-diagnosing a farm that contains a PI as negative. However, neither cut-off can be considered ideal. The predicted probability curve in Fig. 3b is generated for the same seventeen herds that underwent ROC curve analysis and shows how the probability of detecting a herd containing a PI increases as the number of Ab positive YS out of ten tested increases.

### Bulk milk PCR analysis

Not all dairy farms had BM PCR results at this stage of the study - this was simply due to the fact that the test was not offered by the AHPA at the start of farm recruitment. With the exception of Farm 29, all farms returning a positive BM PCR result in Fig. 1 had PI animals identified; Farms 38 & 40, where Farm 38 had a milking PI animal yet Farm 40 did not. There were also six farms which were BM PCR negative and of these, three (Farms 18, 37 & 20) contained PIs none of which were of milking age. The ages of the PI animals identified on these farms (and throughout this study) are discussed in Booth and Brownlie 2012 [32].

**Fig. 4:**
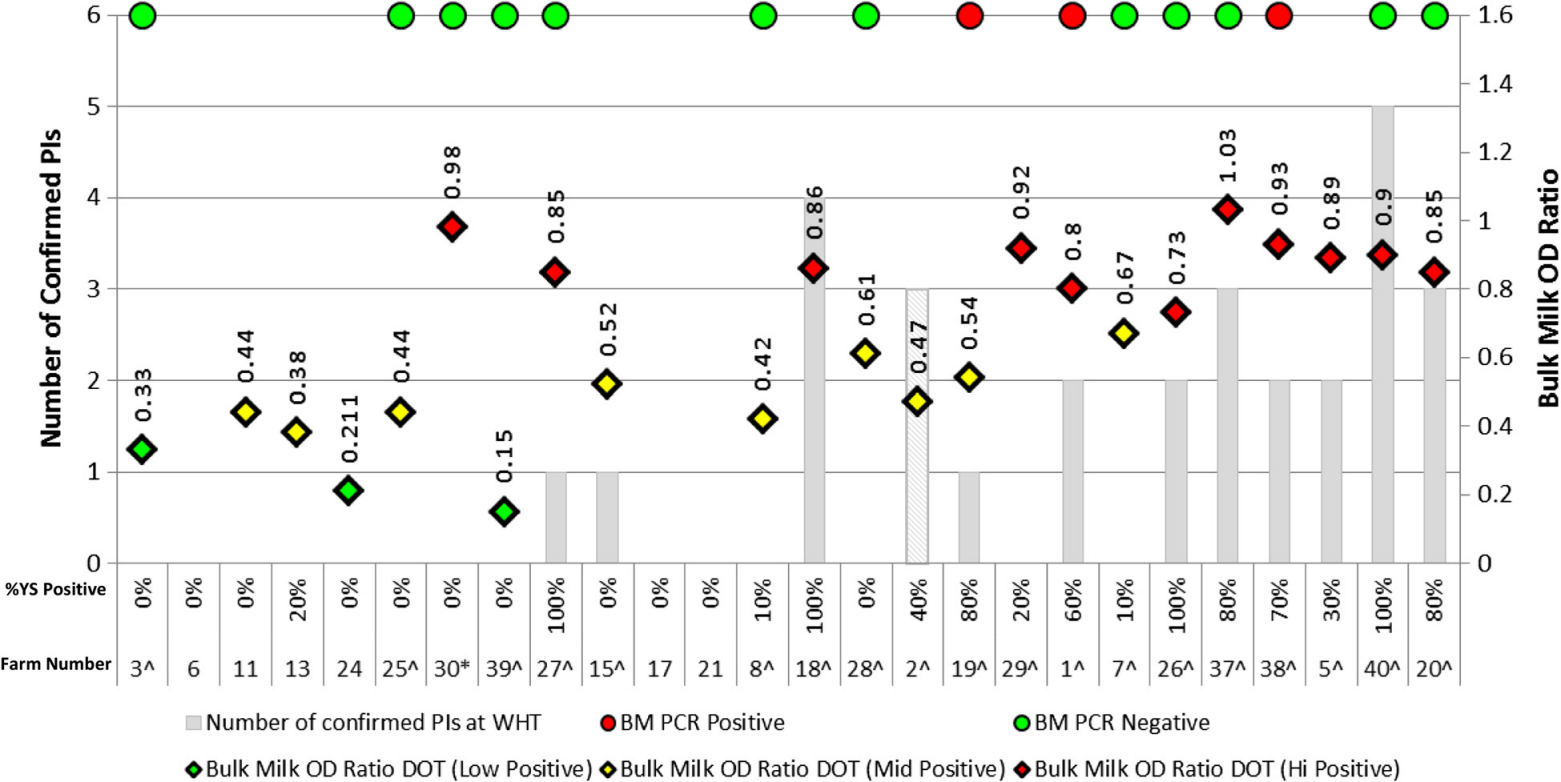
The results of simulated initial herd screens of the 19 herds that undertook WHTs and the Year 2 screens of the remaining 7 herds. The figure illustrates the results of the simulated initial herd screens for the 19 herds that undertook WHTs and the Year 2 screens for the 7 herds that did not. ^indicates that the farm underwent whole herd testing. The left y-axis displays the number of PIs identified at the WHT (where performed) depicted for each farm by the solid bars – the Farm 2 bar is hashed to represent in-utero PI animals. For the 7 farms that did not undertake WHTs because their regular surveillance did not indicate exposure, it was assumed that no PIs were present. The right y-axis displays the BM Ab OD ratio. Along the x -axis, each column represents an individual farm stating both the percentage of antibody positive youngstock (YS) at the Check Test (* all farms submitted ten animals except Farm 30 where only 9 animals of appropriate age were available) and the Farm number. (Graph Key attached to the bottom of Fig. 4)

At this point, Farm 29 is worth highlighting since it returned a positive BM PCR result, a high positive BM Ab result and 5/10 animals Ab positive at the Check Test yet no PIs were identified on the premises either at the WHT or in the testing following this. The delay between the initial herd screens and WHTs to identify PI animals is shown in Table 1 and ranges from 1–19 months a mean interval between initial screens and WHTs of 8 months. The delay for Farm 29 was 12 months between the initial screens presented in Fig. 1 and the WHT to identify PI animals. The impact of these delays between initial screens and what was actually found at the WHT could be considerable and therefore the initial screens presented in Fig. 1 may not accurately reflect herd status at the time the WHT was conducted. For this reason, further data was collected from the farms presented in Fig. 1 so that for those farms that underwent a WHT, the equivalent of an initial screen was generated for that day meaning that the delay between the initial screen and the WHT was effectively 0 days. The age groups of the randomly selected YS are detailed in Table 1 and for most farms, it was possible to select animals from within the 9–12 month range. The YS selected for Farms 18 and 37 were just outside of the 9–12 month range whilst for Farms 8, 15, 25, 26 and 38 it was necessary to extend the range to 7–13 months. These data are presented in Fig. 4. Farms 6, 11, 13, 17, 21, 24, & 30 did not undergo WHT since the results of their regular surveillance (BM Ab, BM PCR and Check Tests) did not justify testing all stock. These farms are included in Fig. 4 and the data presented for each are the year 2 Check Test results which coincided with a quarterly BM Ab and yearly PCR test (where available).

### Bulk milk antibody analysis without delay

**Fig. 5:**
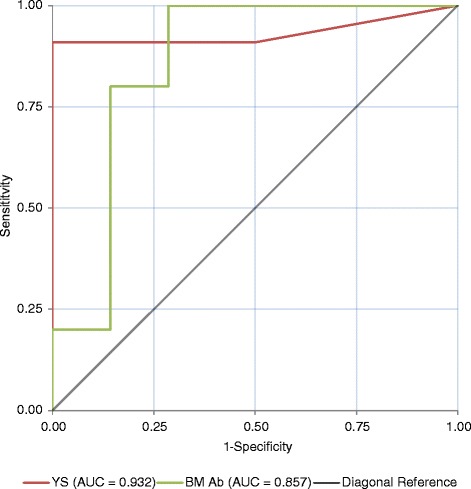
ROC curves demonstrating the performance of BM Ab and YS Check Tests as predictors of the presence of a PI animal(s) where there is no delay between the initial herd screen and the WHT. The ROC curves were generated from the seventeen dairy farms in Fig. 4 with full initial herd screens and WHT results. YS = Youngstock check testing and BM Ab = Bulk milk antibody testing. Farm 2 is considered negative for BM Ab analysis and positive for YS analysis. AUC = Area under curve. Coordinates of the curves are given in Table 3

In the 23 dairy farms represented in Fig. 4 high positive BM Ab results occur on nine infected farms and two that are BVDV-free (Farms 29 & 30), mid positive BM Ab results occur on six BVDV-free farms, Farm 2 and two infected farms (Farms 15 & 19). Finally, three BVDV-free dairy farms in Fig. 4 now return low positive BM Ab results. On Farm 2, the dairy herd was still un-exposed at this point hence should be considered a negative herd when assessing BM Ab. Analysis of BM Ab results from the 17 dairy herds that undertook WHTs produced the ROC curve in Fig. 5, the coordinates of which (Table 3) demonstrate that the optimal BM Ab OD ratio cut-off for distinguishing whether a herd contained a PI animal or not occurs at 0.7 OD ratio units. This also coincides with the AHPA cut off for a high positive result using this test. With this dataset, a sensitivity and specificity of 80.00 % (44.39–97.48 %) and 85.71 % (42.13–99.64 %) respectively (95 % confidence intervals quoted) was achieved. The predicted probability curve in Fig. 3c shows better distinction using BM Ab OD ratio at this cut off compared to Fig. 3a.

**Table 3:** Coordinates of the ROC curves in Fig. 5

Test result variables	Positive if greater than or equal to a or b^#^	Sensitivity	1 - Specificity
^a^Number of youngstock antibody positive out of ten tested without delay	−1.00	1.000	1.000
	.50	.909	.500
	1.50	.909	.167
	2.50	.909	0.000
	3.50	.818	0.000
	5.00	.727	0.000
	6.50	.636	0.000
	7.50	.545	0.000
	9.00	.273	0.000
	11.00	0.000	0.000
^b^Bulk milk antibody OD ratio without delay	−.8500	1.000	1.000
	.2400	1.000	.857
	.3750	1.000	.714
	.4300	1.000	.571
	.4550	1.000	.429
	.4950	1.000	.286
	.5300	.900	.286
	.6050	.800	.286
	.7000	.800	.143
	.7650	.700	.143
	.8250	.600	.143
	.8550	.500	.143
	.8750	.400	.143
	.8950	.300	.143
	.9100	.200	.143
	.9250	.200	0.000
	.9800	.100	0.000
	2.0300	0.000	0.000

^#^ The smallest cutoff value is the minimum observed test value minus 1, and the largest cutoff value is the maximum observed test value plus 1. All the other cutoff values are the averages of two consecutive ordered observed test values

### Check test analysis without delay

Analysing the farms in Fig. 4 using the cautious cut off (defined earlier) of >1/10 Ab positive youngstock results in the false positive misclassification of Farms 13 & 29 and the false negative misclassification of Farm 15. Analysing the Check Test results from the 17 farms produces the ROC curve in Fig. 5, the coordinates of which (Table 3) indicate that an optimal cut-off point occurs at 2.5/10 animals with a sensitivity and specificity of 90.9 % (58.72–99.77 %) and 100 % (54.07–100 %) respectively (95 % confidence intervals quoted). Utilising this cut-off would result in the false negative misclassification of one farm (Farm 15) from the 17 analysed in Fig. 5. The predicted probability curve in Fig. 3d illustrates an improved distinction compared to the tests explored in Fig. 3a-c.

### Bulk milk PCR analysis without delay

At this point all farms that returned a positive BM PCR in Fig. 4 had PIs identified (Farms 1, 19 & 38) where Farms 1 & 38 contained milking PIs, but Farm 19 did not. Five out of the twelve farms that returned BM PCR negative results also contained PI animals at the time the BM was sampled (Farms 20, 26, 27, 37 & 40) however, none of the PI animals on these farms were of milking age.

These results indicate that when herd screening is performed with no delay between that and the WHT to identify PI animals, the sensitivity and specificity of both the BM antibody and the YS cohort screens for determining PI presence in a herd are improved. Furthermore, the predictive probabilities of both tests improve when the delay between screening and WHTs is removed.

## Discussion

This manuscript presents BM Ab, BM PCR and YS Ab (Check Test) data from twenty six working farms that were recruited to a pilot BVDV eradication programme. Although there is general agreement in the current literature that BM Ab levels and youngstock Check Tests are appropriate ways to assess BVDV status at the herd level [20, 25–27, 35, 36], there remains some confusion about their reliability and the impact of historic infection and vaccination. We discuss these issues in the context of our study herds below.

The recruited herds were predominantly located in Somerset, UK and the large proportion of dairy herds in the study population reflects the region from which herds were sampled. The average study herd size appears large when compared to the average herd size of 120 animals quoted by Defra for the Taunton area at the time of recruitment [37]. However, the Defra figure may be falsely low as this will include small holdings and in the authors’ experience, the herd sizes represented here are typical of the range of farms in this region.

Within the analyses conducted, the wide 95 % confidence intervals quoted throughout highlight that there is a degree of uncertainty surrounding the results and thus the figures should be used with caution. This is largely due to the small sample size of 17 herds which underwent statistical analysis. Despite these potential limitations, the improvements demonstrated in sensitivity and specificity and also in the ROC and probability curves by removing the delay between screening a herd and acting on those results are substantial and worth discussion. Of the 26 herds that were recruited, only 17 were suitable for statistical analysis since BM and YS samples were collected in addition to individual animal samples to determine the BVDV status of all animals on this subset of farms. Two farms that had conducted a WHT were deemed unsuitable for inclusion in the statistical analysis as ten animals were not available for the initial YS screens and are included in Figs. 1 and 4. The seven remaining farms did not undergo WHTs and were therefore excluded from the statistical analysis, but are presented for completeness in Figs. 1 and 4. Whilst we believe that the regular surveillance would have detected infection on these farms over, and beyond, the course of the study period analysed here, it remained an assumption that they were BVDV free and so it would have been inappropriate to influence the ROC, probability, sensitivity and specificity analyses on this basis. This point is especially pertinent when we consider that Farm 15 returned a mid positive BM Ab titre at the WHT and also negative YS results in both the initial Check Test and the simulated Check Test from the WHT yet a PI was present throughout the period. This was primarily due to herd dynamics and is discussed in relation to this herd’s results below.

### The Use of BM Ab Tests for determining Herd BVDV Status

BM Ab tests provide an initial, rapid and cost effective assessment of the BVDV status in dairy cattle at the herd level. It is largely accepted that high BM Ab levels correlate with a high probability of the presence of a PI. However, we found several instances where herd BVDV status would have been incorrectly classified if based on an interpretation of the BM Ab result alone. The sensitivity and specificity of the test throughout this study was 80.00 and 85.71 % respectively however, the probability curves indicate that it is difficult to accurately define a cut-off point where we can say with reasonable certainty that a herd does or does not contain a PI animal. The removal of the delay between the BM Ab screening and further investigations does improve the confidence that one can have in the interpretation of the test but as a sole screening method, the usefulness of BM Ab remains limited in many circumstances. We know that both historic BVDV infections and herd vaccination can have a significant effect on correct BM Ab interpretation [33, 38] and both of these factors are likely to have played a significant role in the test results and interpretation here.

BVDV antibodies following a natural infection may persist for 3 or more years in an individual animal [7, 34]. When one considers that BM Ab assesses all milking animals including the oldest in the herd, it explains why it can take in excess of 1000 days for BM Ab to show any real decrease following the removal of PI animals [38]. Booth et al. 2013 [33] demonstrated that Farms 13 & 24, which were BVDV-free farms that neither vaccinated nor experienced active BVDV infection during the study period experienced a slow yet consistent decrease in the BM Ab titre; in this type of herd BM Ab can be a sensitive surveillance test for monitoring for disease incursion into the milking group since an increase in Ab titre can be considered of significance. Vaccination can also confound interpretation of BM Ab results further. With the exception of Farms 13, 24 & 39, all BVDV-free dairy herds in this study vaccinated during the period data was collected (Table 1) and all began the study with mid to high BM Ab titres (Fig. 1). In the longitudinal analysis of BM Ab trends by Booth et al. 2013 [33], BVDV-free vaccinating herds did not demonstrate a clear decrease in BM Ab. The fact that the majority of the study herds were utilising BVDV vaccines, in combination with the likelihood that many of them may have experienced historic infection and thus contain older seropositive animals may explain the higher level of misclassification of false positive farms observed with the BM Ab tests compared to YS Check Tests. In practice, careful thought needs to be given to the application and timing of BM Ab testing in herds utilising BVDV vaccines and the use of this test may be deemed inappropriate in some systems. This is discussed in further detail by Booth and et al. 2013 [33].

Whilst vaccination and historic infection would explain those farms that returned high positive BM Ab results in the absence of PIs, it is more difficult to explain those herds (Farms 15 and 19) that returned only mid-positive BM Ab results in Fig. 4 when PI animals were present within the herd. For these farms, the reasons are likely due to the location of the PI animal(s) within their herds:

- For Farm 15, the lack of a high positive BM Ab result is harder to explain since the PI was of milking age. She was however a poor animal and often separated from the milking herd in a hospital pen due to illness.
- Farm 19 contained a PI animal of approximately 1 year of age and yet returned a mid positive BM Ab result. Upon initial interpretation, it may simply be possible that the PI did not have contact with the milking herd, however, analysis of the BM PCR results bring this interpretation into question since the herd was positive on BM PCR testing. It is difficult to provide an explanation for this combination of results especially given that at the initial screen for Farm 19 (conducted 5 months prior to the WHT), the herd returned a high positive BM Ab result (Fig. 1).

Farm 2 highlights the need for the practitioner to consider the epidemiological units that make up the herd structure in detail both before testing and before coming to a decision on the status of the herd based on the results of the tests. At both the initial herd screen and at the point of the WHT, the milking herd on Farm 2 had not been recently exposed to BVDV, hence the mid positive result. If a single assessment had been performed on a bulk milk sample, and decision reached that the herd was uninfected, then infection in the heifers would have gone unnoticed until subsequent BM Ab titres had increased. Our Check Test results indicated that there was likely exposure in the heifer group at the calf rearer and that this was almost certainly due to the biosecurity breakdown involving mixing with animals from another farm. The return of three heifers carrying PI foetuses to the main farm in combination with the farmers reluctance to cull PI animals once identified and the failure to engage the owner of the other animals at the heifer rearer in the scheme eventually resulted in infection of the main herd and an increase in the BM Ab titre to a high positive (data not shown). This farm is discussed in more detail by Booth and Brownlie 2012 [32].

### The use of young stock (YS) check tests for determining herd BVDV status

The real value of YS antibody tests is that young animals will be seronegative if unexposed and maternally derived Ab (MDA) have waned; if they do have antibodies demonstrating an active immune response (in contrast to MDA), they will have been infected only within their short lifetime (e.g. 9 months). That would indicate a recent, or present, infection on the farm. Assessing animals at, or after, 9 months of age is a valuable time to initiate a YS screen as MDA will have has waned and it is typically pre-vaccination.

YS below six months of age should not be selected for Check Testing since MDA are highly likely to be present. Chamorro et al. 2014 [39] demonstrated that in extreme cases, BVDV MDA can take 11 months to wane and that MDA are commonly present up to 6 months of age; hence have the potential to confound interpretation of the test. In the authors’ experience, testing animals at least 9 months of age, especially in beef suckler herds where colostral transfer of MDA is more reliable and higher quality than in dairy systems, provides a safer margin for ensuring that MDA is not present.

Whilst the majority of the herds in this study did vaccinate, they only initiated the primary course of vaccine prior to first service. The result was that we were able to assess the YS cohorts in these herds without the influence of vaccine. There is the possibility on Farms 8 and 25, that some of the older YS selected from the WHT in order to simulate a Check Test on that day could have been vaccinated as they were at the older end of the 7–13 month range, but in all the other herds, this was unlikely to have influenced the results. In this study, we achieved both a high sensitivity and specificity when assessing the ability of the Check Test to determine whether PI animals were present in the seventeen study herds analysed when the Check Test was simulated from the WHT results. Furthermore, the probability curves displayed an increasing ability to predict the presence of a PI animal within the study herds at the suggested cut off of 2.5/10 animals Ab positive once the delay between screening and the WHT had been reduced. Whilst we have sampled randomly within the animals tested at the WHT in order to generate the ‘without delay’ Check Test, it is important to mention that in the field, careful thought should be given to the contact of the cohort(s) tested with the rest of the herd and this is discussed in the context of Farm 15 below. Previous epidemiological studies have shown that when two or fewer YS of the ten tested are Ab positive, there are unlikely to be PIs present on a farm [27–29] and the optimal cut off from this manuscript of 2.5/10 YS Ab positive supports this finding, even in larger UK herds. The authors would urge readers to use some caution when applying this cut-off value in those herds that return 1/10 or 2/10 positive animals since this may be the start of seroconversion in a group, detection of a declining Ab response to vaccine or MDA. The most effective way to distinguish this is to re-test the same animals at least 28 days later to determine whether seroconversion is underway.

Farm 15 was consistently classified as negative when interpreting YS Check Tests when, in fact, it contained one adult PI animal. The explanation is that the PI animal was bought in as a heifer and, after taking considerable time to get in calf, finally produced a dead calf that was quickly removed. All calves born on Farm 15 were routinely raised on a different unit to adult stock and thus the adult PI animal never had contact with young animals and did not produce a live PI calf that was mixed with the YS. This again highlights the importance of considering the mixing of groups within a farm unit. If the cohorts selected for Check Tests have had reasonable exposure to the rest of the herd, the results may be extrapolated to provide an indication of herd status. However, cohorts of animals reared as isolated groups (or even in some cases as separate herds until first calving) are not suitable for selection for Check Tests to determine whole herd status but only group status. Furthermore, Farm 15 highlights the importance of testing purchased stock however, this animal was purchased before Farm 15 was recruited and was therefore only detected once the study commenced.

In a herd with a high positive BM Ab titre due to historic infection and/or vaccination, sampling cohorts of YS may be the only way to accurately obtain an up to date assessment of herd status and the predicted probability and ROC curves in Figs. 3d and 5 respectively support that this should provide a relatively accurate analysis. In beef herds, monitoring antibody levels in YS cohorts is the only method of assessing the herd hence it is re-assuring that this test performs well. In traditional cow:calf suckler units, Check Tests are potentially much more reliable than in dairy units since there is a greater degree of mixing of animals thus separate epidemiological groups are less likely to occur.

### The use of bulk milk PCR for determining herd BVDV status

BM PCR provided a highly sensitive and cost-effective test for adult PI animals contributing to the milk tank. Unfortunately, our PCR data is incomplete as the test was not widely available at the time of recruitment of many farms.

Over the course of the study, our limited BM PCR results did identify 5 farms with a positive test; on two of these farms (Farms 1 & 38), PIs were found within the milking herd. On Farm 40, no PIs of milking age were identified and only two older animals had left the milking herd in the time between screening and the WHT. Whilst it is possible that either/both of these culled animals had been PI, another potential hypothesis for the positive BM PCR could be that a number of pregnant animals were carrying PIs and thus the level of virus circulating in the milking herd was sufficient to create detectable levels of viral RNA in the bulk milk. On Farm 19 a positive BM PCR was also returned at the whole herd test, but no milking PI animals were identified, nor were further PIs beyond the initial one identified in the youngstock so it would seem unlikely that this could be due to pregnant animals carrying PIs in this instance and may simply be due to contact between the milking animals and YS group containing the PI on this unit. This concept would be supported if the matching milk sample had returned a high positive Ab titre, but as described above, it is difficult to reconcile why this was not the case and a mid positive titre was returned. In contrast, Farm 29 returned a positive BM PCR result yet no PIs were identified at the WHT. On this farm it is highly likely that the delay in conducting a WHT (12 months) resulted in the death or removal of any PIs prior to identification. The fact that 5/10 YS on Farm 29 tested Ab positive at the initial screen and that the herd had a high positive BM Ab titre support the conclusion that PIs had been present on the farm.

It is important to note that BM PCR can only test those animals contributing to the bulk tank on the day the sample was taken. Our results show that some of the herds that did contain PIs did not return a BM PCR positive test result. PIs later identified on these farms had not contributed to the bulk tank. For this reason, provided the upper limit of milking contributors to a sample, as recommended by the testing laboratory, is not exceeded, a negative BM PCR is a reliable indication that any animals contributing to the BM tank, are not PI. A negative result is not, however, a reliable indication that there is not a PI(s) animal elsewhere in the herd.

### The effect of delays between herd screens and whole herd testing

Apart from the 19 month delay between screening and WHTs on Farm 39, most herds were investigated within 8 months of their initial screen. Farm 39 consistently appeared BVDV-free in the regular surveillance conducted and so there was no urgency to perform a WHT in this herd so perhaps it skews the recorded delays unnecessarily. The mean interval is not ideal but in part reflects the reality in practice where results are awaited, reported to the farmer and then a period of discussion and organisation, often avoiding harvesting or silaging, has to occur in order to arrange whole herd testing. If the delay between screening the herd and taking action based on those results is prolonged, the results of the initial screens may not accurately reflect the BVDV status of the herd in question. The data presented here reflects this and in all cases shows an increase in the diagnostic accuracy and relevance of the screens once these delays are removed.

Farms 7, 8, and 29 had delays between initial screens and WHT of 9, 3 and 12 months respectively. Based upon the original YS criteria of >1/10 YS Ab positive indicating an infected herd all were classified as infected. Despite this, no PIs were identified on these three farms during the course of the study (Fig. 1). Farm 8 had a relatively low number of YS seropositive at the initial screen (2/10) which declined further to 1/10 YS Ab positive at the WHT three months later and so is unlikely to have been an infected herd. Whilst no PIs were found, it is highly likely that both farms 29 & 7 were infected due to the number of seropositive YS detected at the initial Check Test; 6/10 and 5/10 YS Ab positive for each farm respectively (Fig. 1). An infected status is further supported for Farm 29 since it returned a positive BM PCR result at the initial screen (Fig. 1). Due to the delays in conducting WHTs on these two farms, it is possible that any PIs had unknowingly been removed from these units prior to undertaking WHTs since both farms show less evidence of BVDV exposure in Fig. 4. Figure 4 demonstrates that, based on Check Tests, herd screens performed closer to the WHT would have indicated that herds 7 & 29 did not contain PI animals at the point of the WHT since their results were 1/10 and 2/10 YS Ab positive respectively (Fig. 4) – this is below the cut off of 2.5/10 animals indicated in the ROC curve in Fig. 5. The result for Farm 29 is further supported by the fact that it was also BM PCR negative at this stage. These results indicate that significant alterations in herd infection status may have occurred on Farms 7 and 29 in the time between the initial screen and WHT.

On Farms 15 & 27 the delay between the initial screens and whole herd tests was 8 & 9 months respectively. Both farms 15 & 27 bought PI animals shortly before the screens presented in Fig. 1 were performed (data not shown). It is evident that for Farm 27, the initial Check Test was likely performed before enough time had elapsed for seroconversion to have occurred within the group screened since continued surveillance on Farm 27 would have detected the presence of the PI animal, since the YS seroprevalence increased from 0/10 YS Ab positive in Fig. 1 to 10/10 YS Ab positive (Fig. 4). This further highlights the need to develop an ongoing surveillance plan. Farm 15 would however remain undetected through YS surveillance and the reasons for this have been discussed above.

## Conclusions

The data presented indicate that the approach to establishing herd status endorsed by CHeCS [25] is robust. Check Tests, BM Ab and BM PCR, if used and interpreted appropriately are useful tools for establishing a herds BVD status. Minimising the delay between screening a herd and acting quickly on the results obtained will increase their diagnostic accuracy and relevance. Whilst a good starting point, a high positive BM Ab result alone is not enough to classify a herd as infected but it should trigger further investigation. In the same way a mid positive BM Ab result does not definitively mark a herd as BVDV-free. BM Ab can prove a useful surveillance tool in established BVDV free herds with low or negative BM Ab titres however; such results were rarely encountered in this study. For this reason, if the study population is representative of herds in cattle dense areas where there is no systematic control of BVDV and biosecurity is difficult to implement, then low or negative BM Ab samples could be considered rare. In this paper, Check Tests performed with the greatest accuracy when diagnosing herd BVD status; only one farm was misclassified when using YS screens (Fig. 4). BM PCR may be used at the same point of the initial screen of YS and BM antibody although, on cost grounds, it would be reasonable to postpone this test until Check Test and BM Ab results indicate the likely presence of a PI.

Finally, it is paramount that longitudinal surveillance using a combination of the techniques discussed in this paper is performed on farms wishing to monitor their BVDV status since this allows for changes in status to be detected early thus enabling prompt action in the event of a BVDV incursion.

## Abbreviations

Ab: Antibody
AHPA: Animal Health and Plant Agency
BM: Bulk milk
BVDV: Bovine Viral Diarrhoea Virus
CHeCS: Cattle Health Certification Standards
PCR: Polymerase chain reaction
PI: Persistently infected
ROC: Receiver operating characteristic
WHT: Whole herd test
YS: Youngstock
